# P-1507. Humoral responses to two versus one dose of pneumococcal conjugate vaccine 20 (PCV20) in lymphoma survivors at 3 months follow up

**DOI:** 10.1093/ofid/ofaf695.1691

**Published:** 2026-01-11

**Authors:** Samantha Trager, Roy F Chemaly, Sairah Ahmed, Huifang Lu, Chijioke Nze, Ella Ariza Heredia, Fareed Khawaja

**Affiliations:** MD Anderson Cancer Center, Houston, TX; The University of Texas MD Anderson Cancer Center, Houston, Texas; The University of Texas MD Anderson Cancer Center, Houston, Texas; MD Anderson Cancer Center, Houston, TX; MD Anderson Cancer Center, Houston, TX; The University of Texas MD Anderson Cancer Center, Houston, Texas; The University of Texas MD Anderson Cancer Center, Houston, Texas

## Abstract

**Background:**

Vaccination against Streptococcus pneumoniae could benefit lymphoma survivors by preventing serious infections. However, B cell lymphoma survivors who were previously treated with anti-CD20 therapy may have suboptimal humoral responses to a single dose of pneumococcal vaccination. Our study aims to compare the humoral responses within 3 months from a single dose or two doses of PCV20 in B cell lymphoma survivors in remission and at least 1 year from the last dose of anti-CD20.

**Table 1:**
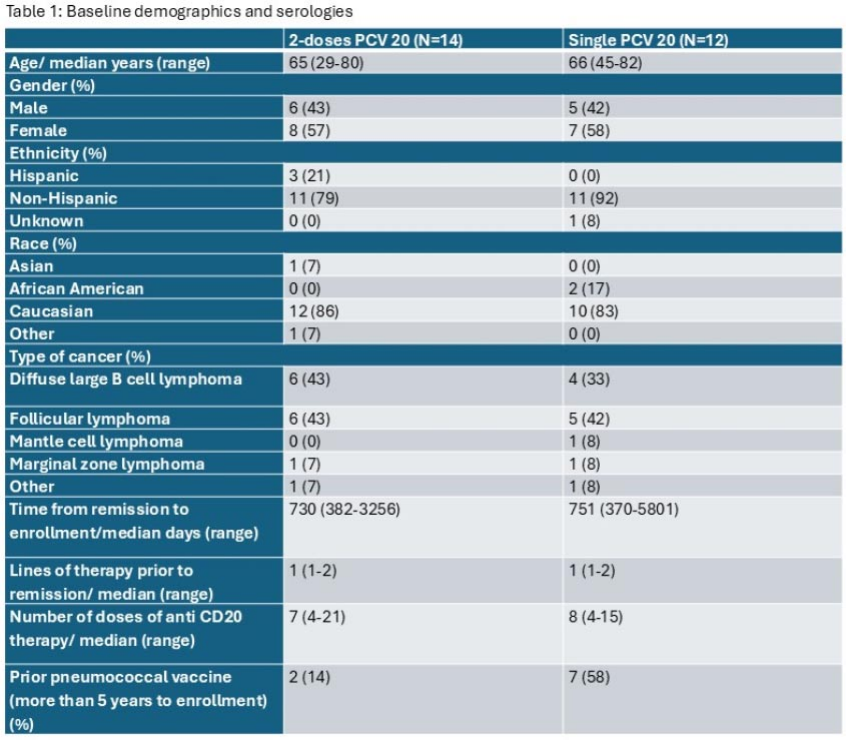
Baseline demographics and serologies

**Figure 1:**
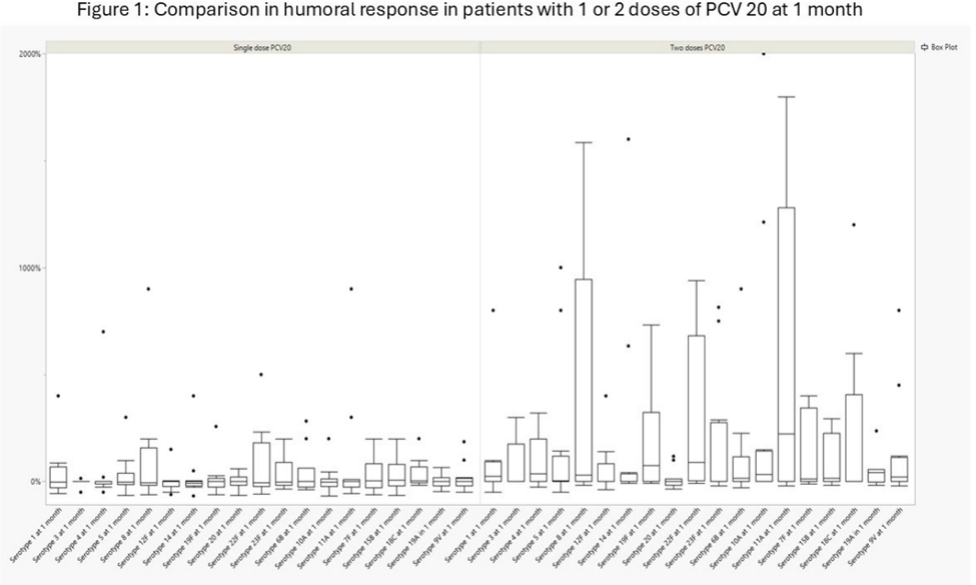
Comparison in humoral response in patients with 1 or 2 doses of PCV 20 at 1 month

**Methods:**

We conducted an open-label randomized trial of PCV20 in B-cell lymphoma survivors who received prior anti-CD20 therapy but no cellular therapy at our center and were in complete remission for at least 1 year. We enrolled 30 patients who were randomized 1:1 to receive 1 or 2 doses of PCV20 (1 month apart). Pneumococcal serologies were measured at enrollment, 1 month and 3 months after the initial vaccination. We compared humoral responses at 1 month and 3 months from the first dose and only serotypes included in the PCV20 vaccination were included.

**Figure 2:**
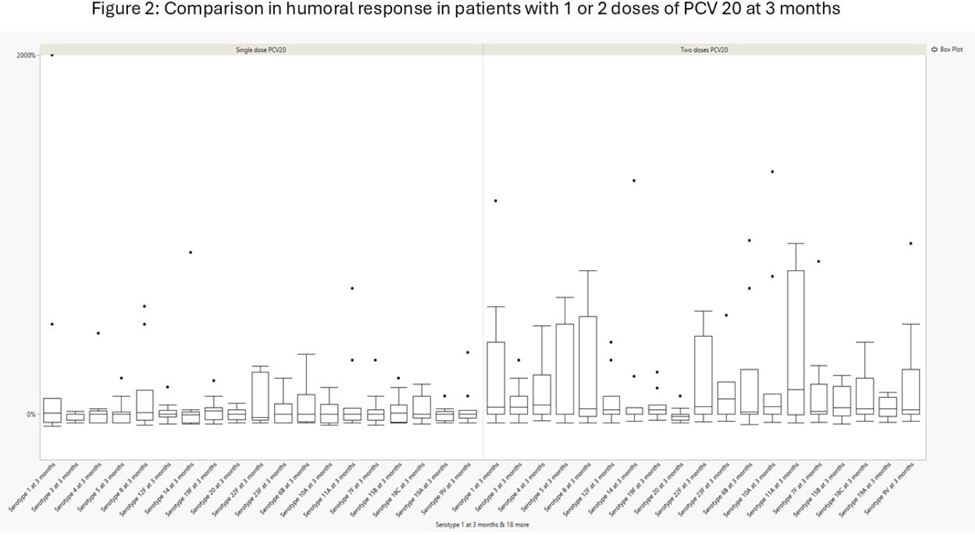
Comparison in humoral response in patients with 1 or 2 doses of PCV 20 at 3 months

**Table 2:**
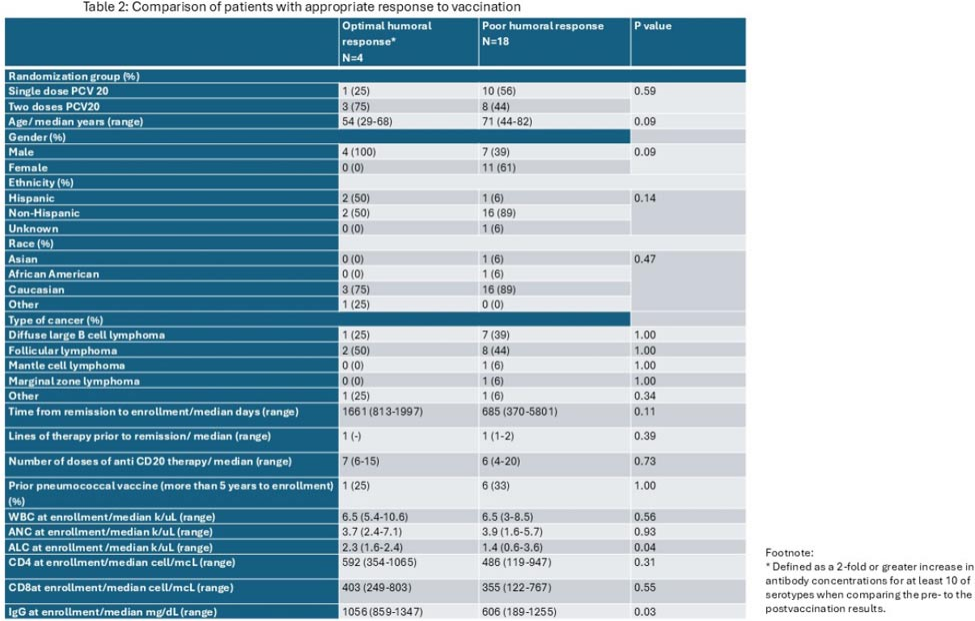
Comparison of patients with appropriate response to vaccination Footnote: * Defined as a 2-fold or greater increase in antibody concentrations for at least 10 of serotypes when comparing the pre- to the postvaccination results.

**Results:**

Out of the 30 randomized patients, 4 withdrew from trial, 14 received 2 doses of PCV20, and 12 received 1 dose of PCV 20 (Table 1). One patient did not undergo serologic testing at baseline and 3 patients did not at months 1 and 3. Overall, there was a greater response in patients who received 2 doses of PCV20 at 1 and 3 months (Figure 1 and 2). On univariate analysis, the difference at 1 month was significant for serotypes 3, 14, 19F, 22F, 10A, 11A and 9V. At 3 months, only serotypes 3 had a statistically significant difference. Patients with higher IgG levels and absolute lymphocyte count (ALC) at time of enrollment had greater humoral responses to PCV20 vaccination (Table 2).

**Conclusion:**

We report an overall greater humoral response to 2 doses of PCV20 compared to 1 in lymphoma survivors at 1 and 3 months from vaccination. IgG levels and ALC were associated with humoral responses.

**Disclosures:**

Roy F. Chemaly, MD, MPH, FIDSA, FACP, FESCMID, ADMA Biologics: Advisor/Consultant|AiCuris: Advisor/Consultant|AiCuris: Grant/Research Support|Ansun Biopharma: Advisor/Consultant|Ansun Biopharma: Grant/Research Support|Assembly Bio: Advisor/Consultant|Astellas: Advisor/Consultant|Eurofins Viracor: Advisor/Consultant|Eurofins Viracor: Grant/Research Support|Genentech: Grant/Research Support|Gilead: Advisor/Consultant|InflaRX: Advisor/Consultant|IntegerBio: Advisor/Consultant|Karius: Advisor/Consultant|Karius: Grant/Research Support|Merck/MSD: Advisor/Consultant|Merck/MSD: Grant/Research Support|Moderna: Advisor/Consultant|Oxford Immunotec: Advisor/Consultant|Pfizer: Advisor/Consultant|Shionogi: Advisor/Consultant|Takeda: Advisor/Consultant|Takeda: Grant/Research Support|Tether: Advisor/Consultant Fareed Khawaja, MBBS, Eurofins Viracor: Grant/Research Support|MERCK: Grant/Research Support|Symbio: Grant/Research Support

